# “Just (Don't) do It?” Stakeholder Perspectives on Physical Activity Interventions in Adult Acute Mental Health Wards: A Qualitative Thematic Analysis

**DOI:** 10.1111/inm.70342

**Published:** 2026-09-07

**Authors:** Michelle Glascott, Philip Hodgson, Wendy Hope, Nicola Clibbens, Luke Aston, Laura Fleming, Alison Innerd, Michael Graham

**Affiliations:** ^1^ Cumbria, Northumberland, Tyne & Wear NHS Foundation Trust Tyne UK; ^2^ Tees, Esk and Wear Valleys NHS Foundation Trust York North Yorkshire UK; ^3^ York St. John University York North Yorkshire UK; ^4^ Department of Nursing and Midwifery Northumbria University Tyne UK; ^5^ Durham Recovery College Durham UK; ^6^ School of Health and Life Sciences Teesside University Middlesbrough North Yorkshire UK

**Keywords:** acute care, exercise therapy, inpatients, mental health nursing, qualitative research

## Abstract

Adult acute mental health wards operate under sustained pressure, characterised by high patient acuity, workforce shortages and a focus on risk management that can limit opportunities for therapeutic engagement. Although physical activity is increasingly recognised as beneficial for mental and physical health, there remains limited evidence to guide its implementation within acute inpatient settings. The purpose of this study was to generate practice‐relevant insights to inform the design and implementation of feasible physical activity interventions in adult acute mental health wards. A qualitative study was conducted using focus groups across two National Health Service mental health trusts in England. Thirty participants took part, including mental health nurses, multidisciplinary staff and people with lived experience of acute ward care. Data were analysed using reflexive thematic analysis within a multidisciplinary and lived‐experience‐informed research team. Three interrelated themes were identified. Barriers to implementation reflected the interaction between patient acuity, medication effects, restrictive practices, workforce pressures and ward organisation. Enablers and opportunities included nursing leadership, flexible and relational delivery approaches, staff participation and active involvement of patients in the design of activities and care planning. Participants also highlighted the importance of evaluation approaches that balance feasibility with meaningful patient‐centred and ward‐level outcomes, including staff wellbeing and ward atmosphere. Physical activity was perceived as both desirable and achievable when adapted to the realities of acute ward environments. These findings address an important evidence gap and can inform the development of context‐sensitive, recovery‐oriented physical activity interventions aligned with acute inpatient mental health nursing practice.

## 1 Introduction

Admission to an adult acute mental health ward (‘acute ward’) is indicated when a person experiencing a severe mental health crisis requires 24 h care to manage risk while assessment and treatment are initiated (Davidson 2021). Over the past three decades, mental health policy internationally has prioritised alternatives to inpatient crisis care, contributing to concerns that acute wards have been comparatively neglected and increasingly positioned as a last resort for those deemed too risky for community provision (Bhugra et al. 2017; Perkins et al. 2023).

Contemporary acute wards operate under sustained pressure, characterised by reduced bed capacity, high patient acuity and chronic staffing shortages (King's Fund 2024a). These conditions are associated with high levels of violence and sexual assault affecting both patients and staff (Brooker and Tocque 2024), creating environments that are often antithetical to therapeutic engagement. Although structured therapeutic activities can improve ward atmosphere, patient experience and reduce restrictive practices (Foye et al. 2020), their delivery is difficult to sustain in volatile settings with rapid patient turnover and limited staffing (Care Quality Commission (CQC) 2017).

Physical activity interventions in inpatient mental health care represent a growing area of research; however, evidence has predominantly been generated within longer‐stay settings, particularly psychiatric rehabilitation and secure services (Martland et al. 2021; Roden‐Lui et al. 2025; Keel et al. 2025). Although acute wards share some structural characteristics with these settings, including multidisciplinary staffing and high rates of compulsory detention, they differ substantially in function and context. Acute wards are characterised by severe mental health crisis, high patient acuity, rapid admission and discharge cycles and significant workforce pressures (King's Fund 2024a). These factors create a uniquely volatile therapeutic environment that may influence both the feasibility and effectiveness of physical activity interventions. Consistent with this, Martland et al. (2023) reported lower uptake and higher attrition in studies conducted on acute wards, while Graham et al.'s (2025) acute‐specific systematic review identified very few eligible studies internationally and substantial methodological limitations. Together, these findings highlight the need for research focused specifically on the acute ward context.

## 2 Background

In the UK, admissions to acute mental health wards increased by 24% between 2016 and 2023, with most admissions occurring under compulsory detention (King's Fund 2024a). Average length of stay is approximately 39 days (Centre for Mental Health 2024) and pressure on bed availability contributes to delays in access to care, resulting in patients presenting with increasing levels of acuity and risk (Department of Health and Social Care 2018; King's Fund 2024a). Acuity is defined by the Health Services Safety Investigations Body (HSSIB 2024, 3) as ‘how unwell a patient is and how much care and support they require’.

While acute ward care is delivered by multidisciplinary teams, mental health nurses and support staff provide the majority of continuous, front‐line care (Davidson 2021). They are disproportionately exposed to the consequences of system pressure, with up to 78% of UK inpatient mental health nurses reporting regular exposure to violence and threats (Weltens et al. 2021), a pattern mirrored internationally (Jang et al. 2022). Persistent staffing shortages, high vacancy and turnover rates and reliance on temporary staff further exacerbate these challenges (King's Fund 2024b), with inexperienced nurses increasingly required to manage high‐risk situations in highly volatile environments (Ford et al. 2024; HSSIB 2024).

These conditions are associated with increased use of restrictive practices, including restraint, rapid tranquilisation and seclusion (NHS Digital 2023). Reducing restrictive interventions is an international priority (LeBel et al. 2014), given their association with physical and psychological harm, escalation of conflict and impeded recovery (Bowers 2014; Sustere and Tarpey 2019). Therapeutic activity is a recognised preventative strategy (Hallett and McLaughlin 2022); however, activities are frequently withdrawn during periods of heightened risk, reinforcing cycles of containment and restriction (Bowers 2014).

Physical activity is an effective intervention in reducing symptoms of depression, anxiety, psychosis and substance use disorders (Ashdown‐Franks et al. 2020) and as an adjunct to pharmacological and psychological treatments can enhance outcomes for depression, suicidality and serious mental illness (Kvam et al. 2016; Sylvia et al. 2010). Individuals with serious mental illness (SMI) experience substantial physical health inequalities and significantly reduced life expectancy (up to 20 years), largely due to preventable physical conditions (Stubbs et al. 2018). While pharmacological treatments offer small to modest symptom relief, they may exacerbate cardiometabolic risk (Vancampfort 2015) and do little to address these inequalities. Physical activity, by contrast, has demonstrated benefits for both physical health and cognitive functioning (Ashdown‐Franks et al. 2019), yet remains marginal within routine inpatient mental health care (Lederman et al. 2017).

Guided by principles of complex intervention development (Skivington et al. 2021), this study aimed to generate practice‐relevant insights from mental health nurses, patients, families and multidisciplinary team members to inform the design and implementation of feasible physical activity interventions within adult acute mental health wards.

## 3 Methods

### 3.1 Design

A qualitative study using reflexive thematic analysis, as described by Braun and Clarke (2006), was conducted using focus groups across two NHS mental health trusts in England. The study was informed by a pragmatic philosophical orientation and principles of participatory action research, foregrounding experiential knowledge to inform practice‐relevant intervention development (Allemang et al. 2022; Cornish et al. 2023). The study is reported in accordance with the Consolidated Criteria for Reporting Qualitative Research (COREQ) guidelines (Tong et al. 2007) (Data S1).

### 3.2 Researcher Positionality and Reflexivity

The research team comprised mental health nurses, lived experience (LEXP) researchers, physiotherapists and academic researchers, bringing diverse professional and experiential perspectives to the study. Several team members had direct experience of working on or receiving care within acute mental health wards, while others contributed expertise in physical activity and qualitative research methods. The team included researchers of different genders.

Reflexivity was actively embedded throughout the research process. Team members engaged in ongoing reflective discussion to consider how their professional roles, lived experience and assumptions might shape data collection, interpretation and theme development. Collaborative analysis and reflexive dialogue across clinical, academic and lived experience perspectives were used deliberately to enhance analytical depth, challenge singular interpretations and support transparency in decision‐making (Braun and Clarke 2019).

### 3.3 Ethical Considerations

Ethical approval was obtained from a UK Research Ethics Committee in April 2023 (REC reference: 23/EE/0089). All participants received written information about the study and were encouraged to ask questions before providing written consent. Given the potential for discussion of distressing experiences associated with acute inpatient care, researchers remained attentive to signs of distress and pre‐ and post‐focus group support was offered to all participants.

### 3.4 Study Setting

The study was conducted across two large NHS mental health trusts in England, each serving mixed urban and rural populations with high levels of socioeconomic deprivation and physical and mental health need. They diverged in staffing configurations, ward environments and operational policy.

### 3.5 Recruitment and Sampling

Purposive sampling was used to recruit both acute ward staff and people with recent lived experience (within the past 5 years) of acute ward care. All regular staff working on acute wards at participating sites were eligible. Recruitment was conducted via posters displayed in staff areas and distribution via ward email lists. Staff who expressed interest were invited to attend a focus group held within their employing trust.

People with lived experience were recruited through NHS trust involvement banks and via posters displayed at a local recovery college. Interested individuals were invited to participate in a focus group held at their local recovery college.

A target sample of 24 participants was set, with approximately equal representation from each site and a minimum of 10 staff and 10 LEXP participants. Focus groups comprised between four and eight participants to support inclusive discussion and psychological safety (Salkind 2010).

### 3.6 Data Collection

A semi‐structured topic guide was co‐developed by clinical and LEXP researchers and informed by findings from a recent systematic review (Graham et al. 2025). Topics included experiences of physical activity on acute wards, perceived barriers and facilitators to implementation and perspectives on evaluating physical activity interventions (Data S2).

Focus groups were co‐facilitated by one LEXP researcher and one clinical researcher. Data were collected between July and October 2023. Facilitators recorded brief reflective notes following each focus group. All focus groups were audio‐recorded, transcribed verbatim and anonymised prior to analysis.

### 3.7 Data Analysis

Data analysis followed Braun and Clarke's (2006) six‐phase reflexive thematic analysis. Analysis was conducted collaboratively by clinical and lived experience (LEXP) researchers, supported by academic team members with qualitative expertise.

Three transcripts were randomly selected and independently analysed through familiarisation, initial coding and preliminary theme development. The research team then met to share reflexive interpretations, discuss convergence and divergence across codes and develop an initial thematic framework. This framework was used to support analysis of the remaining transcripts, with themes iteratively refined through ongoing team discussion.

In keeping with reflexive thematic analysis, saturation was not treated as a fixed or purely data‐driven endpoint. Rather, data collection and analysis continued until the research team judged that sufficient depth and diversity of perspectives had been achieved to support coherent, meaningful theme development in relation to the study aims. This judgement was informed by ongoing reflexive engagement with the data and multidisciplinary analytic discussion (Braun and Clarke 2019).

Reflexivity was embedded throughout the analytic process through collaborative discussion between academic, clinical and lived experience researchers. Differing interpretations of the data were actively explored to challenge assumptions and refine theme development. For example, early interpretations of participant accounts concerning psychotropic medication emphasised reduced motivation as a barrier to physical activity. Discussion with lived experience researchers highlighted that participants were often describing sedation and reduced physical agency rather than unwillingness to engage. This informed refinement of coding and theme descriptions. Reflexive dialogue across the research team supported analytical depth, transparency and confirmability throughout the analytic process, culminating in the final naming and reporting of themes.

## 4 Findings

### 4.1 Overview

Six focus groups were conducted across two NHS mental health trusts, comprising 30 participants: 10 people with lived experience (LEXP) of acute ward care and 20 staff members (Table 1). Staff participants represented a range of professional roles, including mental health nursing, occupational therapy, physiotherapy, exercise therapy, activity coordination and healthcare support.

**TABLE 1:** Summary of focus group participation.

Focus group ID	Study site	Facilitators	Participants	Number of participants (*n*)	Focus group duration (minutes)	Mode of delivery
1: LEXPFG1	1	MG and WH	LEXP	2	60	Face‐to‐face
2: LEXPFG2	1	MG and WH	LEXP	4	60	Face‐to‐face
3: SFG3	1	MG and WH	Staff	8	90	Face‐to‐face
4: LEXPFG4	2	PH and LA	LEXP	4	120	Face‐to‐face
5: SFG5	2	PH and LA	Staff	6	120	Video call
6: SFG6	2	PH and LA	Staff	6	120	Video call

Although the original sampling plan sought equal representation, staff interest exceeded expectations and all interested staff were accommodated to capture a broad range of ward‐based perspectives. Participants were grouped by role (LEXP or staff); however, substantial convergence in perspectives was evident across groups.

Analysis generated three interrelated themes considered central to the development of acceptable and feasible physical activity interventions in acute ward settings: (1) barriers to implementation, (2) enablers and opportunities and (3) measuring impact (Table 2).

**TABLE 2:** Thematic map of themes and sub‐themes.

Themes	Sub‐themes
1. Barriers to implementation	Patient factors Operational factors
2. Enablers and opportunities	Operationalising Encouraging Involving
3. Measuring impact	Patient focussed Staff wellbeing and Ward atmosphere

### 4.2 Theme 1: Barriers to Implementation

**FIGURE 1:**
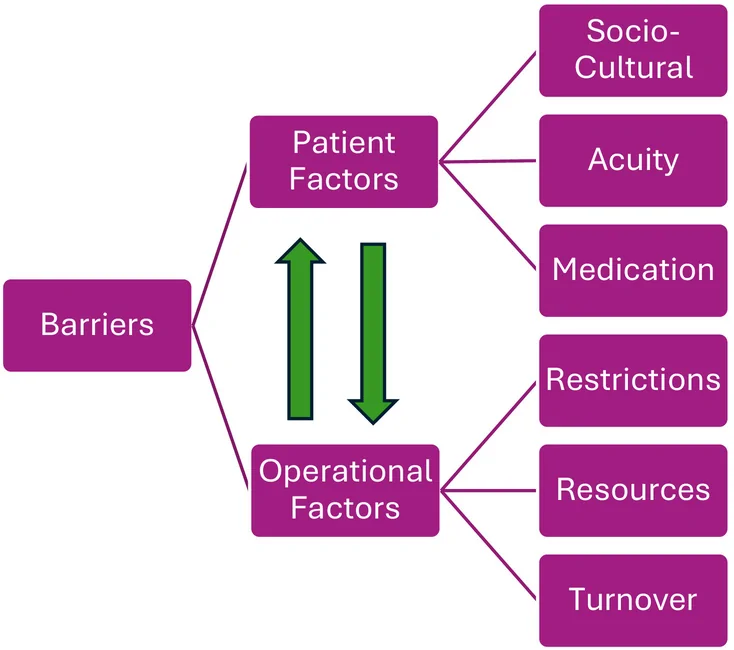
Barriers to Implementation.

Barriers were framed by participants as arising from both patient‐related and operational factors, which were described as dynamically interacting rather than discrete (Figure 1).

#### 4.2.1 Patient Factors

Participants identified socio‐cultural context, acuity and medication effects as key influences on engagement with physical activity.

Staff described many patients as entering acute wards with long‐standing social disadvantage and limited prior exposure to health‐promoting behaviours, shaping expectations and confidence around physical activity.

*‘We have to understand our demography as well.…I think what it taps into… generational inactivity. And whether it comes to, like, healthy eating or drug use, or alcohol use or anything… We're potentially trying to challenge very ingrained behaviours’*. [SFG5]

High levels of distress and risk at admission were consistently reported as limiting capacity for participation. Both staff and LEXP participants described environments characterised by fear, interpersonal conflict and collapse in functioning, which were perceived as incompatible with physical activity during early admission. Sedating medications, while acknowledged as clinically necessary, were experienced as reducing energy, motivation and physical capability, further constraining engagement.

*‘By the time people come to us, they're in a state of total collapse and crisis, otherwise they wouldn't make it through the door.’* [SFG6]

*‘Sometimes the effects of the medication can put you in a state of mind where you're not able to go to the gym or take part in exercise. When I was on Aripiprazole I was, like, really lethargic. Drooling’*. [LEXPFG2]

#### 4.2.2 Operational Factors

Operational barriers reflected the priority given to risk management within acute wards. LEXP participants described extensive restrictions on movement and activity, experienced as frustrating and disempowering.

*‘I was, like, sectioned and I wasn't allowed off the ward or anything like that – I had to make do with just walking round the corridor of the ward. Backwards and forwards…’*. [LEXPFG2]

Staff similarly reported that safety imperatives and risk aversion frequently resulted in the cancellation of non‐pharmacological interventions, including physical activity.

Workforce pressures were described as a critical barrier. Staff shortages, competing clinical demands and reliance on ‘firefighting’ approaches limited opportunities for therapeutic engagement.

*‘And safety is, you know, clearly at the top of the tree. If you put all your resources to target that particular area, … then what else, you know, falls away? You know, there's so many risks that have to be considered… So, you know, the culture again just goes… Just don't. Don't do it’*. [SFG6]

Restricted access to facilities, particularly outside weekday hours, further curtailed activity provision. Rapid patient turnover compounded these challenges, with staff describing insufficient time to build trust, motivate engagement or sustain interventions.

### 4.3 Theme 2: Enablers and Opportunities

**FIGURE 2:**
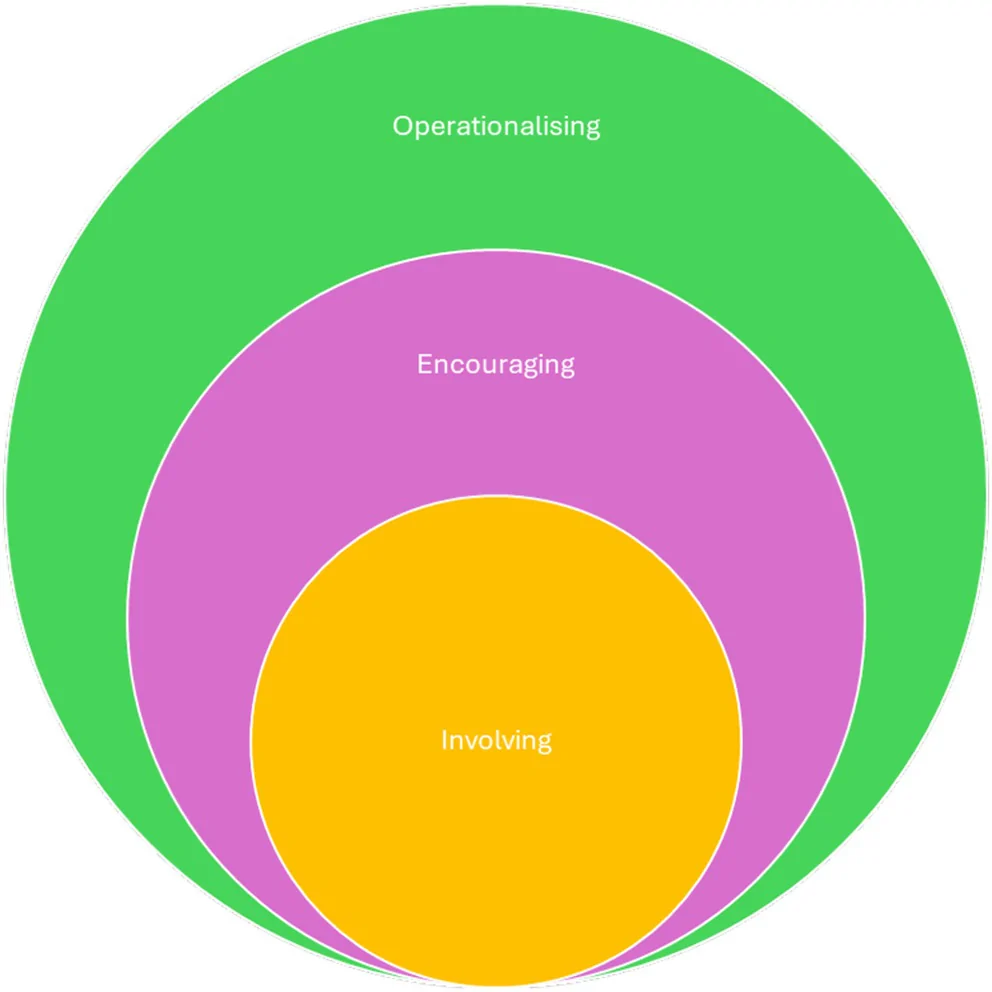
Enablers and opportunities.

Despite these constraints, participants identified clear, practice‐based strategies for enabling physical activity on acute wards (Figure 2). Three interconnected sub‐themes were identified: operationalising, encouraging and involving.

#### 4.3.1 Operationalising

Leadership was positioned as central to implementation success. Staff emphasised that visible managerial engagement legitimised physical activity as part of ward culture, while executive‐level endorsement was viewed as necessary to secure sustainability beyond individual wards.

*‘Staff buy in was entirely dependent on whether the ward manager took part, really. And then, when she did, everybody was on the floor doing it. Whereas when she didn't…’*. [SFG3]

*‘If it's an absolute buy in at executive level, and then that's communicated down as, you know, this is something that we want to see. Make it happen. There's strategy behind it’*. [SFG5]

Participants described ambiguity regarding responsibility for delivering physical activity. While staff debated whether specialist or generalist roles were required, LEXP participants consistently favoured delivery by trained exercise professionals. Some staff sought to reconcile these tensions by broadening definitions of physical activity to include informal, ward‐based movement that could be facilitated across professional roles, particularly during weekends.

*‘Because there's a difference between physical activity and structured, planned exercise… So, things like walking groups and badminton or… You know, a game of table tennis, or whatever, on the ward on the weekend – there's no reason why you need an exercise therapist or anybody like that in that role. So, they're the kind of activities that could be offered on the weekend’*. [SFG3]

#### 4.3.2 Encouraging

Encouragement strategies ranged from formal education to subtle integration within routine care. Participants highlighted the importance of reframing physical activity to avoid triggering negative associations with “exercise” and to promote inclusive, enjoyable alternatives.

*‘Some people have a negative look on it [exercise]… Whereas, like, if we promote, like, other activities that they might enjoy – whether it's gardening or, like, anything to get them moving… Then it can change the way they, like, perceive the words, physical activity*’. [SFG5]

LEXP participants noted that inadequate communication about available activities limited uptake, particularly when information was outdated or inconsistent.

*‘A bit more information, things that attract people's attention, you know. On what's available, on what days. But it must be updated’*. [LEXPFG4]

Staff described embedding physical activity within everyday interactions—such as walking during one‐to‐one sessions—as a low‐burden strategy that reduced formality and increased acceptability.

*‘I like to do my nursing one‐to‐ones off the ward, because you don't get interrupted, … ‘shall we just go and have a little walk round the grounds and get some fresh air?’*. [SFG3].

Visible, communal activities were also described as generating curiosity and social momentum, facilitating gradual engagement.

*‘They want to know what's going on – oh, what are they doing in that room?’ At the end, ‘oh what were you doing in there?’ And then obviously they start talking about it themselves. And then, ‘oh well, next time, will you give me a shout’ and… You build up from there’*. [SFG5]

#### 4.3.3 Involving

Across all groups, involvement was viewed as fundamental. Participants emphasised co‐designing activities through community meetings and supporting personalised plans that offered choice and autonomy. Involvement was framed as particularly meaningful within restrictive environments, enabling patients to regain a sense of control and ownership.

*‘… Talking about… it at community meetings… and getting the community to maybe design what they want for the next week.’* [SFG3]

*‘You can choose how you exercise. How often you exercise… You can have control over’*. [LEXPFG1]

### 4.4 Theme 3: Measuring Impact

Participants identified the need for evaluation approaches that balanced feasibility with meaningful outcome capture, addressing both patient‐centred and ward‐level impacts.

#### 4.4.1 Patient‐Focused Outcomes

Simple, rapid measures of mood, enjoyment and subjective benefit were favoured to minimise burden.

*‘Yeah, maybe like a tick‐box system from one to five – something like that. Something very basic and easy*’. [LEXPFG2].

Participants also suggested drawing on routinely collected clinical and physical health data, including medication use, physical health markers and readmission rates.

*‘When you're in hospital, you get your bloods and all that taken a lot. So, if there's differences in there, that will be a good indicator’*. [LEXPFG4].

However, quantitative metrics alone were viewed as insufficient. Both staff and LEXP participants advocated for incorporating patient narratives to contextualise outcomes and illuminate mechanisms of change.

*‘The readmission rate is fine, but the problem is the reasons why people are readmitted’*. [SFG6]

*‘Just asking the patient themselves how they feel. If it's having any improvements on their mental health’*. [LEXPFG2].

#### 4.4.2 Staff Wellbeing and Ward Milieu

Staff participants with prior experience of ward‐based physical activity described perceived improvements in morale, relationships and team cohesion.

*‘When we do physical activity on the ward, you see the improvements in the morale of the team. Both staff and patients’*. [SFG3].

Participants suggested that routinely collected safety and incident data could be explored alongside activity provision to assess potential associations with ward atmosphere and conflict.

*‘Could you look at like the incident data and whether there was, like, more activity that day and less incidents, yeah’* [SFG5].

## 5 Discussion

This study adds to ongoing critiques of adult acute mental health wards as environments that prioritise medicalised risk containment at the expense of therapeutic engagement (CQC 2017). Consistent with previous research, participants described persistent challenges to implementing non‐pharmacological interventions, including high patient turnover, limited staffing and the profound distress of the patient group (Raphael et al. 2021). However, this study extends existing literature by illuminating how these constraints are actively negotiated by staff and patients and by identifying practical, context‐sensitive strategies to support physical activity in acute ward settings.

Participants' accounts reflected the complex interplay between patient‐related factors—such as socio‐economic disadvantage, trauma histories, substance use and the sedating effects of psychotropic medication—and operational pressures on wards. While these factors are well‐documented in the literature (Davidson 2021; King's Fund 2024c), participants emphasised how their convergence in acute settings creates conditions in which physical activity is easily perceived as unfeasible or unsafe. The resulting emphasis on rapid pharmacological containment, often delivered within inexperienced and under‐resourced nursing teams (Ford et al. 2024), contributes to a cycle in which restrictive practices dominate and therapeutic activities are deprioritised (Bowers 2014).

Despite these challenges, participants consistently framed physical activity as both desirable and achievable, provided that delivery was adapted to the realities of acute inpatient care. This finding aligns with previous work conducted in broader inpatient settings (Martland et al. 2021; Ball et al. 2022), while underscoring the distinct pressures that differentiate acute wards from rehabilitation or forensic environments. Unlike longer‐stay settings, acute wards are characterised by severe psychological distress, rapid patient turnover and competing risk‐management priorities, limiting opportunities to establish therapeutic routines and sustain engagement (King's Fund 2024a). Our findings suggest that interventions developed in rehabilitation or secure services may therefore require substantial adaptation before implementation in acute inpatient settings.

Leadership emerged as a central enabler. Participants highlighted that in risk‐averse environments, nursing staff require explicit permission and visible endorsement to prioritise non‐pharmacological interventions, particularly during periods of heightened acuity. This supports previous findings that leadership engagement is crucial in sustaining cultural change on acute wards (Raphael et al. 2021). Where managers modelled participation and positioned physical activity as legitimate care, staff confidence and engagement increased. Conversely, ambivalence from senior nurses or ward managers undermined implementation, reinforcing perceptions that such activities were dispensable (McCann and Bowers 2005).

Tensions regarding professional roles reflected wider workforce pressures, yet participants demonstrated pragmatic flexibility. While people with lived experience expressed a clear preference for specialist‐led interventions, staff participants broadened definitions of physical activity to include informal, nurse‐facilitated movement embedded within routine care. This reframing aligns with the ‘Making Every Contact Count’ approach (Public Health England 2016) and supports evidence that promoting physical activity is a shared responsibility across professional roles (Lekka et al. 2021). Importantly, this perspective avoids over‐reliance on scarce specialist resources while maintaining therapeutic intent.

Engagement strategies proposed by participants centred on reframing physical activity as meaningful, accessible and relational. Educational approaches—particularly when delivered or endorsed by peers with lived experience—were perceived as valuable, but insufficient in isolation. Subtle behavioural strategies, such as incorporating movement into routine nursing interactions or offering visible, communal activities, were viewed as particularly effective in lowering thresholds for participation. These findings resonate with Ball et al. (2022), who described the value of ‘disguised’ activity and with evidence that staff participation can reduce power differentials and strengthen therapeutic relationships in inpatient care (Raphael et al. 2021).

Involvement and co‐production were unanimously identified as essential. Participants emphasised that engaging patients in designing activities and developing personalised plans enabled choice and autonomy within otherwise restrictive environments. This finding aligns with broader evidence demonstrating that co‐designed interventions enhance engagement among people with serious mental illness (Wheeler et al. 2018) and echoes insights from the SPACES study regarding the marginalisation of service user perspectives (Walker et al. 2023). While SPACES focused on community contexts, participants in this study affirmed the relevance—and necessity—of co‐production within acute inpatient care.

The emphasis participants placed on choice, involvement and personalised approaches aligns closely with principles of recovery‐oriented care (Perkins et al. 2023). Acute wards are necessarily restrictive environments where autonomy may be reduced through risk management processes, compulsory treatment and limitations on freedom of movement. Within this context, participants described physical activity as more than a health intervention, viewing it as an opportunity to restore choice, agency and participation. The ability to select activities, influence programme design and engage in meaningful ways was considered particularly important. Physical activity may therefore provide a practical means of embedding recovery‐oriented principles within routine acute inpatient care.

Participants also emphasised the importance of evaluating physical activity beyond symptom reduction alone. While simple quantitative measures were viewed as feasible, participants advocated for triangulating these with patient‐reported experiences to capture meaning and mechanism. This perspective addresses a notable gap identified by Martland et al. (2023), who found that the majority of inpatient physical activity studies privileged clinical outcomes over experiential data. Participants further highlighted the ethical and practical importance of minimising data collection burden on acutely distressed patients and stretched clinical teams—an issue seldom addressed in existing literature.

Participants also described physical activity as a potential means of reducing boredom, agitation and interpersonal conflict on the ward. This is noteworthy, as physical activity is rarely considered a violence‐reduction strategy within acute inpatient care. However, access to meaningful therapeutic activity has been associated with improvements in ward atmosphere and reductions in violence (Foye et al. 2020), while emerging physical activity studies have reported reductions in aggression and emotional distress (Rahman et al. 2022; Keel et al. 2025). Participants' accounts suggest that these effects may arise through multiple pathways, including providing a constructive outlet for distress, reducing boredom and frustration, supporting emotional regulation and creating opportunities for positive social and therapeutic interactions. Similarly, Javid et al. (2024) found that staff and patients perceived participation in a boxing intervention as helping to manage anger, reduce stress and improve control over impulsive behaviours. Together, these findings suggest that physical activity may have benefits that extend beyond individual health outcomes and contribute to safer and more therapeutic ward environments. Finally, participants identified staff wellbeing, ward milieu, incident rates and use of ‘as required’ medication as potentially meaningful, yet under‐explored, outcomes. These data are routinely collected in acute settings and were perceived as offering valuable indicators of system‐level impact without increasing burden. Future research examining these outcomes may provide important insights into the broader value of physical activity interventions for both patients and staff within acute wards.

### 5.1 Strengths and Limitations

This study provides novel insights into stakeholder perspectives on physical activity within adult acute mental health wards, a setting that remains underrepresented within the existing literature. A key strength was the collaboration between academic, clinical and lived experience researchers throughout study design, data collection and analysis. This multidisciplinary approach facilitated reflexive discussion during theme development and helped challenge assumptions, enhancing the depth, credibility and interpretive richness of the findings.

This study has several limitations that should be considered when interpreting the findings. Participants were self‐selected, which may have resulted in over‐representation of individuals with a particular interest in or positive orientation towards, physical activity. However, the inclusion of diverse professional roles and people with lived experience supported a broad range of perspectives.

Participants were recruited from two NHS mental health trusts within a single region of England. While this may limit the transferability of findings to other geographical or organisational contexts, many themes identified are consistent with existing literature from acute inpatient settings elsewhere in the UK.

The use of focus groups may have constrained disclosure of more sensitive or critical experiences; however, separating staff and lived experience groups was intended to support psychological safety and encourage open discussion.

Member checking was not undertaken. In keeping with reflexive thematic analysis, findings are understood as interpretive rather than representational and participant validation was not considered essential to analytic rigour. Rigour was instead supported through collaborative analysis, reflexive dialogue within a multidisciplinary research team and transparent reporting of analytic decisions.

## 6 Conclusion

This study provides practice‐relevant insights into the feasibility of physical activity interventions within adult acute mental health wards, highlighting the dynamic interplay between patient acuity, workforce pressures and ward culture. Findings underscore the importance of nursing leadership, flexible delivery models and co‐produced approaches in supporting non‐pharmacological care within highly risk‐focused environments. By foregrounding perspectives from nurses, people with lived experience and the multidisciplinary team, this study addresses a significant evidence gap in acute inpatient mental health care. Findings suggest that future intervention development and research should prioritise implementation considerations alongside effectiveness outcomes. Interventions that fail to account for acute ward realities, including staffing pressures, patient turnover and competing risk‐management demands, are unlikely to be sustainable irrespective of their clinical efficacy.

### 6.1 Relevance to Clinical Practice

This study provides transferable, practice‐informed insights into implementing physical activity within adult acute mental health wards, a setting internationally characterised by high acuity and workforce pressures. By foregrounding nursing, lived experience and multidisciplinary perspectives, the findings highlight actionable principles for mental health nursing practice—clinical leadership, flexible delivery and co‐production that are applicable across diverse inpatient contexts. These principles support the integration of recovery‐oriented, non‐pharmacological interventions into routine mental health nursing practice, with potential benefits for patient engagement, staff wellbeing and ward milieu.

## Funding

This work was supported by Academic Health Science Network‐ North East and North Cumbria ICB, 967‐22‐23.

## Ethics Statement

Ethical approval was obtained from the Cambridge East Research Ethics Committee: [REC Ref: 23/EE/0089] Integrated Research Application System (IRAS‐321626) on 28 April 2023.

## Conflicts of Interest

The authors declare no conflicts of interest.

## Supporting information


**Data S1:** COREQ checklist.

**Data S2:** Topic guide for focus group.

## Data Availability

The data that support the findings of this study are available on request from the corresponding author. The data are not publicly available due to privacy or ethical restrictions.
