# Supplementary material for: “Just (Don't) do It?” Stakeholder Perspectives on Physical Activity Interventions in Adult Acute Mental Health Wards: A Qualitative Thematic Analysis
Source: Int J Ment Health Nurs. 2026 Sep 7;35(5):e70342. doi: 10.1111/inm.70342 (PMC13550944; doi:10.1111/inm.70342)
Supplement: Supplementary file 1 [file INM-35-0-s001.docx]

**Supplementary Material: COREQ Checklist**

**Consolidated Criteria for Reporting Qualitative Research (COREQ)**
(Tong, Sainsbury & Craig, 2007)

| **No.** | **Item** | **Guide question** | **Reported in manuscript** |
| --- | --- | --- | --- |
| **Domain 1: Research team and reflexivity** |  |  |  |
| 1 | Interviewer/facilitator | Which author conducted the interview or focus group? | Methods – Data Collection |
| 2 | Credentials | What were the researcher’s credentials? | Methods – Researcher Positionality and Reflexivity |
| 3 | Occupation | What was their occupation at the time of the study? | Methods – Researcher Positionality and Reflexivity |
| 4 | Gender | Was the researcher’s gender reported? | Methods – Researcher Positionality and Reflexivity |
| 5 | Experience and training | What experience or training did the researcher have? | Methods – Design; Researcher Positionality and Reflexivity |
| 6 | Relationship established | Was a relationship established prior to study commencement? | Methods – Researcher Positionality and Reflexivity |
| 7 | Participant knowledge | What did participants know about the researcher? | Methods – Participant Information and Consent |
| 8 | Interviewer characteristics | What characteristics were reported about the interviewer/facilitator? | Methods – Researcher Positionality and Reflexivity |
| **Domain 2: Study design** |  |  |  |
| 9 | Methodological orientation | What methodological approach underpinned the study? | Methods – Design |
| 10 | Sampling | How were participants selected? | Methods – Recruitment and Sampling |
| 11 | Method of approach | How were participants approached? | Methods – Recruitment |
| 12 | Sample size | How many participants were included? | Findings – Overview |
| 13 | Non‑participation | How many people refused to participate or dropped out? | Not reported- recruitment was open and voluntary |
| 14 | Setting of data collection | Where was data collected? | Methods – Study Setting |
| 15 | Presence of non‑participants | Was anyone else present besides participants and researchers? | Not reported. |
| 16 | Description of sample | What were the sample characteristics? | Findings – Table 1 |
| 17 | Interview guide | Were questions or prompts provided? | Methods – Data Collection; Supplementary Material |
| 18 | Repeat interviews | Were repeat interviews carried out? | Not applicable |
| 19 | Audio/visual recording | Were interviews recorded? | Methods – Data Collection |
| 20 | Field notes | Were field notes made? | Methods – Data Collection |
| 21 | Duration | What was the duration of interviews or focus groups? | Findings – Table 1 |
| 22 | Data saturation | Was data saturation discussed? | Methods – Data Analysis |
| 23 | Transcripts returned | Were transcripts returned to participants for comment? | Limitations |
| **Domain 3: Analysis and findings** |  |  |  |
| 24 | Number of data coders | How many data coders were involved? | Methods – Data Analysis |
| 25 | Description of coding tree | Was a coding tree provided? | Findings – Table 2 |
| 26 | Derivation of themes | Were themes identified in advance or derived from data? | Methods – Data Analysis |
| 27 | Software | What software was used to manage the data? | Not reported- manual analysis |
| 28 | Participant checking | Were participants asked to provide feedback on findings? | Limitations |
| 29 | Quotations presented | Were participant quotations presented? | Findings |
| 30 | Data and findings consistency | Was consistency demonstrated between data and findings? | Findings and Discussion |
| 31 | Clarity of major themes | Were major themes clearly presented? | Findings |
| 32 | Clarity of minor themes | Were sub‑themes clearly described? | Findings |
