# Supplementary material for: “Just (Don't) do It?” Stakeholder Perspectives on Physical Activity Interventions in Adult Acute Mental Health Wards: A Qualitative Thematic Analysis
Source: Int J Ment Health Nurs. 2026 Sep 7;35(5):e70342. doi: 10.1111/inm.70342 (PMC13550944; doi:10.1111/inm.70342)
Supplement: Supplementary file 2 [file INM-35-0-s002.docx]

**Supplementary Material 2: Topic Guide for Focus Group**

**Introduction**

Housekeeping – toilets, fire safety, refreshments, duration

Group rules – taking turns to talk, don’t have to say anything not comfortable with; please keep things confidential within the group

Distress – experiences can be distressing, supportive environment

Introduction – summary of study

**People’s Experiences**

Experiences of physical activity in general?

Experiences of physical activity and mental health?

Previous expectations and experiences of the availability of physical activity on acute inpatient mental health wards

**Designing the Physical Activity Intervention**

We want to design a physical activity intervention to be delivered in an acute inpatient mental health ward.

What physical activities could be offered on an acute inpatient mental health ward?

What are the barriers and facilitators in delivering a physical activity on an acute inpatient mental health ward?

Who should deliver this?

How should we promote the physical activity with patients?

How shall we encourage participation?

Any other things you want to add about physical activity on acute inpatient mental health wards?

**The impact of the intervention**

How will we know if anything changes for people because they have taken part in the physical activity?
